# Parliament and the Profession

**Published:** 1917-08-25

**Authors:** 


### Parliament and the Profession.

### Civilian and Military Medical Services.

A NUMBER of questions have been put to the representative of the War Office recently as to the economical utilisation of the services of medical men in the Army, and the proper apportionment of available doctors between civil and military duties. With regard to these points, Mr. Macpherson assured Major D. Davies that the War Office does not call up doctors irrespective of civil needs, and that the fullest and best use is made of the doctors serving on the various Fronts. The number of medical men serving as privates in the ranks is unknown, but every inducement is given to them to take commissions in the Royal Army Medical Corps. Further questioned, Mr. Macpherson stated that some little time ago the Secretary of State arranged for certain eminent medical men to visit France for the purpose of satisfying himself that the Medical Services were on a sound and efficient basis. The visit of this committee will take place when the operations now in progress admit of their carrying out useful inquiries.

### Army Medical Advisory Board.

QUESTIONED as to the composition and work of the Medical Advisory Board of the Army Medical Service, Mr. Macpherson stated that there are eleven members of this Board, seven military and four civilian. The civilian members are paid £200 a year each, and two military members receive extra pay of £150 a year, five receive nothing. The Board has sat once since the war started, but all paid members have been constantly consulted and have given very valuable advice throughout the war. Most members are serving in various theatres of war.

### Position of Territorial Medical Officers.

MR. MACPHERSON explained to Sir G. Thomas that Territorial and Reserve medical officers with R.A.M.C. commissions hold a fair share of the administrative posts in the Army. The chief object of the War Office is to employ a man where his abilities will have the best scope, having regard to the fact that a civilian medical man's attainments, however eminent, are of less value in certain high administrative posts in the Army than knowledge of the administration and organisation of the Army as a whole.

### Defective Eyesight and Gas-Masks.

MR. BILLING asked the Under-Secretary of State for War if the wearing of gas-respirators in combination with steel helmets precludes short-sighted men from also wearing their spectacles, and thus renders them blind and helpless in an advance. Mr. Macpherson replied that his attention had not previously been called to the facts brought to notice, but he was consulting the military authorities in France on the point.

### Vaccination Statistics.

MR. HAYES FISHER supplied Mr. Snowden with the following statistics in respect of the working of the Vaccination Act, 1907, in England and Wales in the year 1915, stating that returns for 1916 are not yet available.

| | |
|---|---|
| Declarations of conscientious objectors ... | 293,315 |
| Births ... ... ... ... ... ... ... | 814,527 |
| Primary vaccinations ... ... ... ... | 375,330 |

The figures for Scotland, for the same year, as supplied by Mr. Munro, in response to an inquiry by Mr. Wilkie, were :

| | |
|---|---|
| Declarations of conscientious objectors ... | 35,231 |
| Births ... ... ... ... ... ... ... | 114,181 |
| Children vaccinated ... ... ... ... ... | 61,456 |

### Deaths Through Anti-Typhoid Inoculation.

MR. W. THORNE asked the Under-Secretary of State for War whether he is aware that ten deaths have been officially admitted in the South African Parliament as due to the effects of anti-typhoid inoculation among the troops, and Mr. Macpherson answered that the unfortunate incident was due to the use of untested vaccine, which was found to be contaminated. This vaccine was made in South Africa by the South African Institute of Medical Research, and not by the Army Medical College. The War Office has offered to supply all future requirements of anti-typhoid vaccine for use in South Africa.

### Colonel Carter.

THE Secretary for India informed Mr. Gilbert that Major (temporary Lieutenant-Colonel) Carter should receive brevet promotion to Lieutenant-Colonel. The promotion will be substantially ante-dated. As Colonel Carter is now employed in this country, the question of his future employment in India cannot advantageously be considered at present. The promotion carries no increase in pay, but it means substantial improvement in position in the Service.

